# Cyberbullying Among Adolescents and Children: A Comprehensive Review of the Global Situation, Risk Factors, and Preventive Measures

**DOI:** 10.3389/fpubh.2021.634909

**Published:** 2021-03-11

**Authors:** Chengyan Zhu, Shiqing Huang, Richard Evans, Wei Zhang

**Affiliations:** ^1^School of Political Science and Public Administration, Wuhan University, Wuhan, China; ^2^School of Medicine and Health Management, Tongji Medical College, Huazhong University of Science and Technology, Wuhan, China; ^3^College of Engineering, Design and Physical Sciences, Brunel University London, Uxbridge, United Kingdom

**Keywords:** cyberbullying, children, adolescents, globalization, risk factors, preventive measures

## Abstract

**Background:** Cyberbullying is well-recognized as a severe public health issue which affects both adolescents and children. Most extant studies have focused on national and regional effects of cyberbullying, with few examining the global perspective of cyberbullying. This systematic review comprehensively examines the global situation, risk factors, and preventive measures taken worldwide to fight cyberbullying among adolescents and children.

**Methods:** A systematic review of available literature was completed following PRISMA guidelines using the search themes “cyberbullying” and “adolescent or children”; the time frame was from January 1st, 2015 to December 31st, 2019. Eight academic databases pertaining to public health, and communication and psychology were consulted, namely: Web of Science, Science Direct, PubMed, Google Scholar, ProQuest, Communication & Mass Media Complete, CINAHL, and PsycArticles. Additional records identified through other sources included the references of reviews and two websites, Cyberbullying Research Center and United Nations Children's Fund. A total of 63 studies out of 2070 were included in our final review focusing on cyberbullying prevalence and risk factors.

**Results:** The prevalence rates of cyberbullying preparation ranged from 6.0 to 46.3%, while the rates of cyberbullying victimization ranged from 13.99 to 57.5%, based on 63 references. Verbal violence was the most common type of cyberbullying. Fourteen risk factors and three protective factors were revealed in this study. At the personal level, variables associated with cyberbullying including age, gender, online behavior, race, health condition, past experience of victimization, and impulsiveness were reviewed as risk factors. Likewise, at the situational level, parent-child relationship, interpersonal relationships, and geographical location were also reviewed in relation to cyberbullying. As for protective factors, empathy and emotional intelligence, parent-child relationship, and school climate were frequently mentioned.

**Conclusion:** The prevalence rate of cyberbullying has increased significantly in the observed 5-year period, and it is imperative that researchers from low and middle income countries focus sufficient attention on cyberbullying of children and adolescents. Despite a lack of scientific intervention research on cyberbullying, the review also identified several promising strategies for its prevention from the perspectives of youths, parents and schools. More research on cyberbullying is needed, especially on the issue of cross-national cyberbullying. International cooperation, multi-pronged and systematic approaches are highly encouraged to deal with cyberbullying.

## Introduction

Childhood and adolescence are not only periods of growth, but also of emerging risk taking. Young people during these periods are particularly vulnerable and cannot fully understand the connection between behaviors and consequences (1). With peer pressures, the heat of passion, children and adolescents usually perform worse than adults when people are required to maintain self-discipline to achieve good results in unfamiliar situations. Impulsiveness, sensation seeking, thrill seeking, and other individual differences cause adolescents to risk rejecting standardized risk interventions (2).

About one-third of Internet users in the world are children and adolescents under the age of 18 (3). Digital technology provide a new form of interpersonal communication (4). However, surveys and news reports also show another picture in the Internet Age. The dark side of young people's internet usage is that they may bully or suffer from others' bullying in cyberspace. This behavior is also acknowledged as cyberbullying (5). Based on Olweus's definition, cyberbullying is usually regarded as bullying implemented through electronic media (6, 7). Specifically, cyberbullying among children and adolescents can be summarized as the intentional and repeated harm from one or more peers that occurs in cyberspace caused by the use of computers, smartphones and other devices (4, 8–12). In recent years, new forms of cyberbullying behaviors have emerged, such as cyberstalking and online dating abuse (13–15).

Although cyberbullying is still a relatively new field of research, cyberbullying among adolescents is considered to be a serious public health issue that is closely related to adolescents' behavior, mental health and development (16, 17). The increasing rate of Internet adoption worldwide and the popularity of social media platforms among the young people have worsened this situation with most children and adolescents experiencing cyberbullying or online victimization during their lives. The confines of space and time are alleviated for bullies in virtual environments, creating new venues for cyberbullying with no geographical boundaries (6). Cyberbullying exerts negative effects on many aspects of young people's lives, including personal privacy invasion and psychological disorders. The influence of cyberbullying may be worse than traditional bullying as perpetrators can act anonymously and connect easily with children and adolescents at any time (18). In comparison with traditional victims, those bullied online show greater levels of depression, anxiety and loneliness (19). Self-esteem problems and school absenteeism have also proven to be related to cyberbullying (20).

Due to changes in use and behavioral patterns among the youth on social media, the manifestations and risk factors of cyberbullying have faced significant transformation. Further, as the boundaries of cyberbullying are not limited by geography, cyberbullying may not be a problem contained within a single country. In this sense, cyberbullying is a global problem and tackling it requires greater international collaboration. The adverse effects caused by cyberbullying, including reduced safety, lower educational attainment, poorer mental health and greater unhappiness, led UNICEF to state that “no child is absolutely safe in the digital world” (3).

Extant research has examined the prevalence and risk factors of cyberbullying to unravel the complexity of cyberbullying across different countries and their corresponding causes. However, due to variations in cyberbullying measurement and methodologies, no consistent conclusions have been drawn (21). Studies into inconsistencies in prevalence rates of cyberbullying, measured in the same country during the same time period, occur frequently. Selkie et al. systematically reviewed cyberbullying among American middle and high school students aged 10–19 years old in 2015, and revealed that the prevalence of cyberbullying victimization ranged from 3 to 72%, while perpetration ranged from 1 to 41% (22). Risk and protective factors have also been broadly studied, but confirmation is still needed of those factors which have more significant effects on cyberbullying among young people. Clarification of these issues would be useful to allow further research to recognize cyberbullying more accurately.

This review aims to extend prior contributions and provide a comprehensive review of cyberbullying of children and adolescents from a global perspective, with the focus being on prevalence, associated risk factors and protective factors across countries. It is necessary to provide a global panorama based on research syntheses to fill the gaps in knowledge on this topic.

## Methods

### Search Strategies

This study strictly employed Preferred Reporting Items for Systematic Reviews and Meta-Analyses (PRISMA) guidelines. We consulted eight academic databases pertaining to public health, and communication and psychology, namely: Web of Science, Science Direct, PubMed, Google Scholar, ProQuest, Communication & Mass Media Complete, CINAHL, and PsycArticles. Additional records identified through other sources included the references of reviews and two websites, Cyberbullying Research Center and United Nations Children's Fund. With regard to the duration of our review, since most studies on cyberbullying arose around 2015 (9, 21), this study highlights the complementary aspects of the available information about cyberbullying during the recent 5 year period from January 1st, 2015 to December 31st, 2019.

One researcher extracted keywords and two researchers proposed modifications. We used two sets of subject terms to review articles, “cyberbullying” and “child OR adolescent.” Some keywords that refer to cyberbullying behaviors and young people are also included, such as threat, harass, intimidate, abuse, insult, humiliate, condemn, isolate, embarrass, forgery, slander, flame, stalk, manhunt, as well as teen, youth, young people and student. The search formula is (cyberbullying OR cyber-bullying OR cyber-aggression OR ((cyber OR online OR electronic OR Internet) AND (bully^*^ OR aggres^*^ OR violence OR perpetrat^*^ OR victim^*^ OR threat^*^ OR harass^*^ OR intimidat^*^ OR ^*^ OR insult^*^ OR humiliate^*^ OR condemn^*^ OR isolate^*^ OR embarrass^*^ OR forgery OR slander^*^ OR flame OR stalk^*^ OR manhunt))) AND (adolescen^*^ OR child OR children OR teen? OR teenager? OR youth? OR “young people” OR “elementary school student^*^” OR “middle school student^*^” OR “high school student^*^”). The main search approach is title search. Search strategies varied according to the database consulted, and we did not limit the type of literature for inclusion. Journals, conference papers and dissertations are all available.

**Figure 1:**
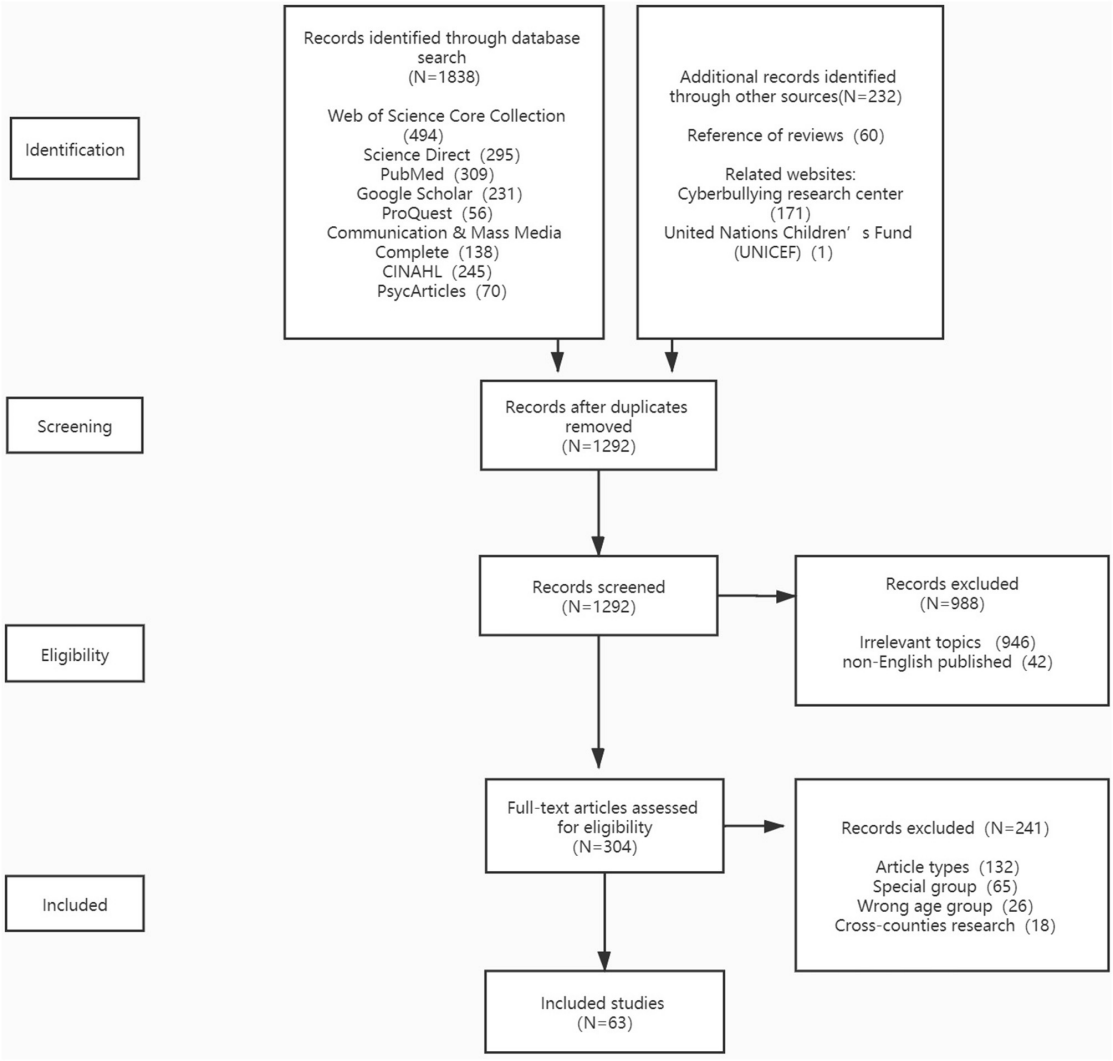
PRISMA flow chart diagram showing the process of study selection for inclusion in the systematic review on children and adolescents cyberbullying.

Specifically, the inclusion criteria for our study were as follows: (a). reported or evaluated the prevalence and possible risk factors associated with cyberbullying, (b). respondents were students under the age of 18 or in primary, junior or senior high schools, and (c). studies were written in English. Exclusion criteria were: (a). respondents came from specific groups, such as clinical samples, children with disabilities, sexual minorities, specific ethnic groups, specific faith groups or samples with cross-national background, (b). review studies, qualitative studies, conceptual studies, book reviews, news reports or abstracts of meetings, and (c). studies focused solely on preventive measures that were usually meta-analytic and qualitative in nature. Figure 1 presents the details of the employed screening process, showing that a total of 63 studies out of 2070 were included in our final review.

Meta-analysis was not conducted as the limited research published within the 5 years revealed little research which reported odds ratio. On the other hand, due to the inconsistency of concepts, measuring instruments and recall periods, considerable variation could be found in research quality (23). Meta-analysis is not a preferred method.

### Coding Scheme

For coding, we created a comprehensive code scheme to include the characteristics. For cyberbullying, we coded five types proposed by Willard (24–26), which included verbal violence, group violence, visual violence, impersonating and account forgery, and other behaviors. Among them, verbal violence is considered one of the most common types of cyberbullying and refers to the behavior of offensive responses, insults, mocking, threats, slander, and harassment. Group violence is associated with preventing others from joining certain groups or isolating others, forcing others to leave the group. Visual violence relates to the release and sharing of embarrassing photos and information without the owners' consent. Impersonating and account forgery refers to identity theft, stealing passwords, violating accounts and the creation of fake accounts to fraudulently present the behavior of others. Other behaviors include disclosure of privacy, sexual harassment, and cyberstalking. To comprehensively examine cyberbullying, we coded cyberbullying behaviors from both the perspectives of cyberbullying perpetrators and victims, if mentioned in the studies.

In relation to risk factors, we drew insights from the general aggression model, which contributes to the understanding of personal and situational factors in the cyberbullying of children and adolescents. We chose the general aggression model because (a) it contains more situational factors than other models (e.g., social ecological models) - such as school climate (9), and (b) we believe that the general aggression model is more suitable for helping researchers conduct a systematic review of cyberbullying risk and protective factors. This model provides a comprehensive framework that integrates domain specific theories of aggression, and has been widely applied in cyberbullying research (27). For instance, Kowalski and colleagues proposed a cyberbullying encounter through the general aggression model to understand the formation and development process of youth cyberbullying related to both victimization and perpetration (9). Victims and perpetrators enter the cyberbullying encounter with various individual characteristics, experiences, attitudes, desires, personalities, and motives that intersect to determine the course of the interaction. Correspondingly, the antecedents pertaining to cyberbullying are divided into two broad categories, personal factors and situational factors. Personal factors refer to individual characteristics, such as gender, age, motivation, personality, psychological states, socioeconomic status and technology use, values and perceptions, and other maladaptive behaviors. Situational factors focus on the provocation/support, parental involvement, school climate, and perceived anonymity. Consequently, our coders related to risk factors consisting of personal factors and situational factors from the perspectives of both cyberbullying perpetrators and victims.

We extracted information relating to individual papers and sample characteristics, including authors, year of publication, country, article type, sampling procedures, sample characteristics, measures of cyberbullying, and prevalence and risk factors from both cyberbullying perpetration and victimization perspectives. The key words extraction and coding work were performed twice by two trained research assistants in health informatics. The consistency test results are as follows: the Kappa value with “personal factors” was 0.932, and the Kappa value with “situational factors” was 0.807. The result shows that the coding consistency was high enough and acceptable. Disagreements were resolved through discussion with other authors.

### Quality Assessment of Studies

The quality assessment of the studies is based on the recommended tool for assessing risk of bias, Cochrane Collaboration. This quality assessment tool focused on seven items: random sequence generation, allocation concealment, blinding of participants and personnel, blinding of outcome assessment, incomplete outcome data, selective reporting, and other sources of bias (28). We assessed each item as “low risk,” “high risk,” and “unclear” for included studies. A study is considered of “high quality” when it meets three or more “low risk” requirements. When one or more main flaw of a study may affect the research results, the study is considered as “low quality.” When a lack of information leads to a difficult judgement, the quality is considered to be “unclear.” Please refer to Appendix 1 for more details.

## Results

This comprehensive systematic review comprised a total of 63 studies. Appendices 2, 3 show the descriptive information of the studies included. Among them, 58 (92%) studies measured two or more cyberbullying behavior types. The sample sizes of the youths range from several hundred to tens of thousands, with one thousand to five thousand being the most common. As for study distribution, the United States of America, Spain and China were most frequently mentioned. Table 1 presents the detail.

**Table 1:** Descriptive information of studies included (2015–2019).

**Study location**	**Number of studies**	**Proportion(%)**
United States of America	14	22
Spain	12	19
China	6	10
Israel	5	8
Turkey	5	8
Canada	4	6
South Korea	3	5
Others	14	22
Total	63	100

### Prevalence of Global Cyberbullying

#### Prevalence Across Countries

Among the 63 studies included, 22 studies reported on cyberbullying prevalence and 20 studies reported on prevalence from victimization and perpetration perspectives, respectively. Among the 20 studies, 11 national studies indicated that the prevalence of cyberbullying victimization and cyberbullying perpetration ranged from 14.6 to 52.2% and 6.3 to 32%, respectively. These studies were conducted in the United States of America (*N* = 4) (29–32), South Korea (*N* = 3) (33–35), Singapore (*N* = 1) (36), Malaysia (*N* = 1) (37), Israel (*N* = 1) (38), and Canada (*N* = 1) (39). Only one of these 11 national studies is from an upper middle income country, and the rest are from highincome countries identified by the World Bank (40). By combining regional and community-level studies, the prevalence of cyberbullying victimization and cyberbullying perpetration ranged from 13.99 to 57.5% and 6.0 to 46.3%, respectively. Spain reported the highest prevalence of cyberbullying victimization (57.5%) (41), followed by Malaysia (52.2%) (37), Israel (45%) (42), and China (44.5%) (43). The lowest reported victim rates were observed in Canada (13.99%) and South Korea (14.6%) (34, 39). The reported prevalence of cyberbullying victimization in the United States of America ranged from 15.5 to 31.4% (29, 44), while in Israel, rates ranged from 30 to 45% (26, 42). In China, rates ranged from 6 to 46.3% with the country showing the highest prevalence of cyberbullying perpetration (46.30%) (15, 43, 45, 46). Canadian and South Korean studies reported the lowest prevalence of cyberbullying perpetration at 7.99 and 6.3%, respectively (34, 39).

**Figure 2:**
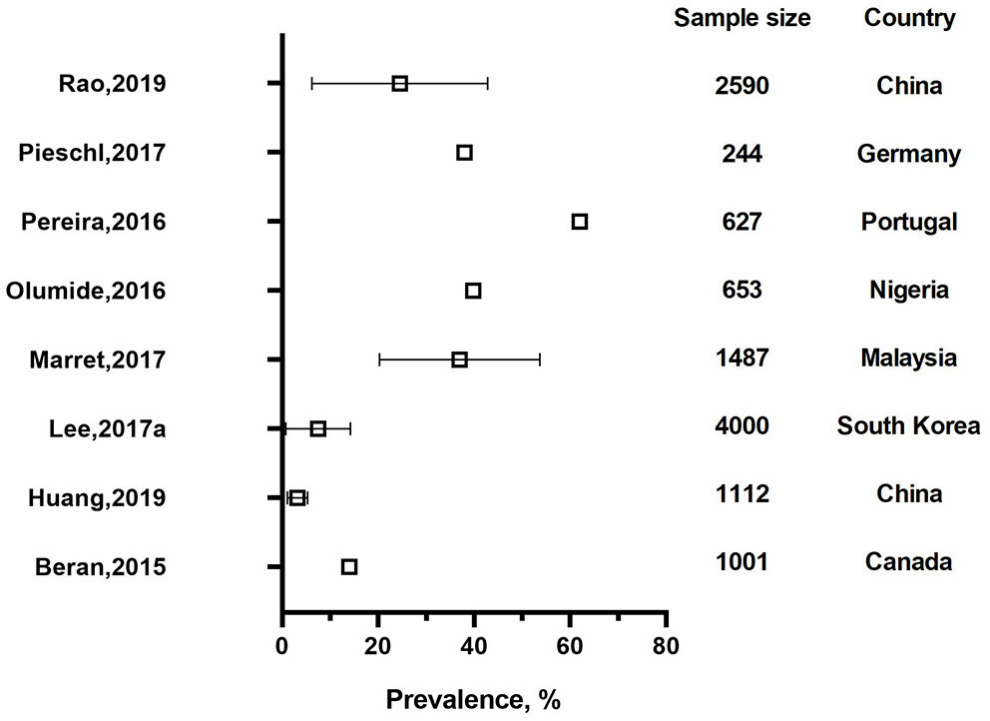
The prevalence of cyberbullying victimization of high quality studies.

**Figure 3:**
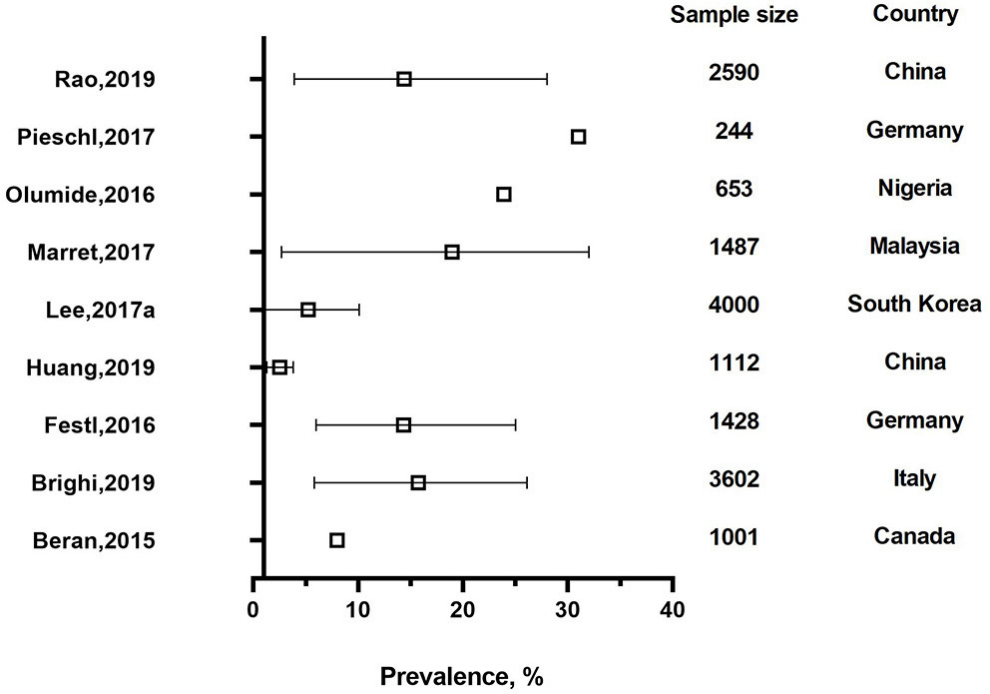
The prevalence of cyberbullying perpetration of high quality studies.

A total of 10 studies were assessed as high quality studies. Among them, six studies came from high income countries, including Canada, Germany, Italy, Portugal, and South Korea (13, 34, 39, 46–48). Three studies were from upper middle income countries, including Malaysia and China (37, 43) and one from a lower middle income country, Nigeria (49). Figures 2, 3 describe the prevalence of cyberbullying victimization and perpetration respectively among high quality studies.

#### Prevalence of Various Cyberbullying Behaviors

**Figure 4:**
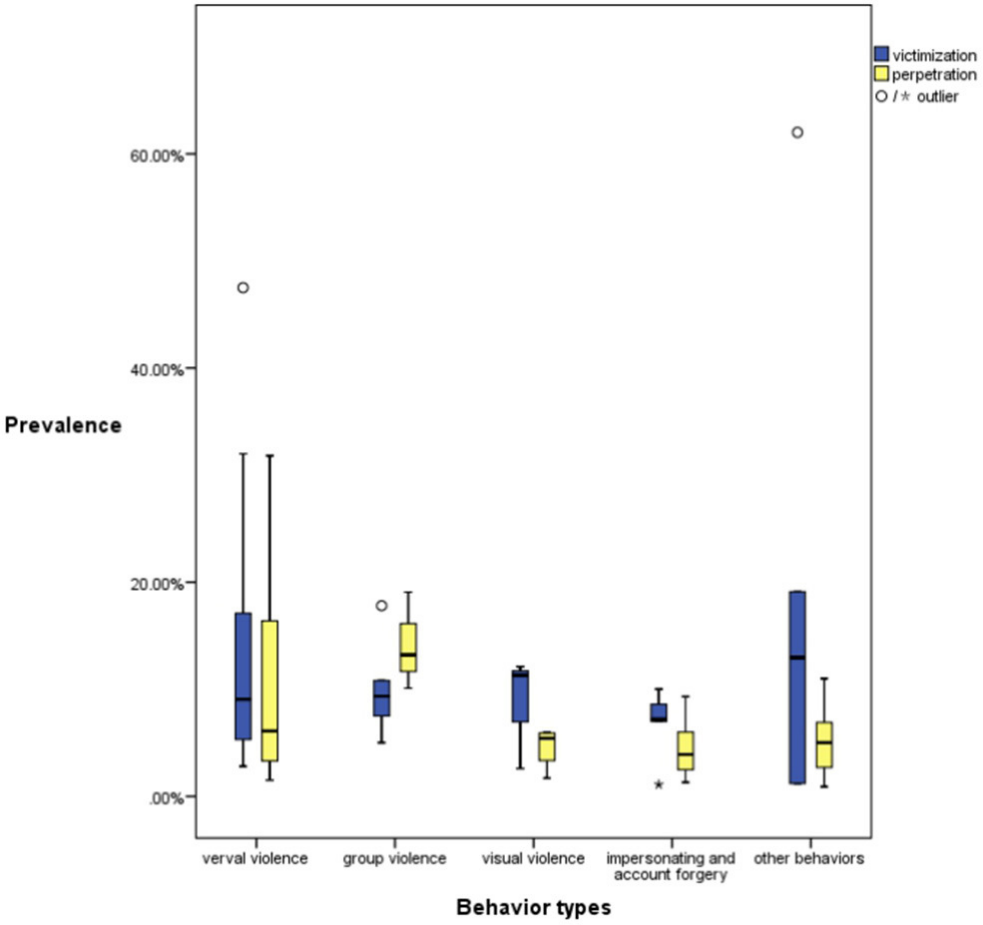
Cyberbullying prevalence across types (2015–2019).

For the prevalence of cyberbullying victimization and perpetration, the data were reported in 18 and 14 studies, respectively. Figure 4 shows the distribution characteristics of the estimated value of prevalence of different cyberbullying behaviors with box plots. The longer the box, the greater the degree of variation of the numerical data and vice versa. The rate of victimization and crime of verbal violence, as well as the rate of victimization of other behaviors, such as cyberstalking and digital dating abuse, has a large degree of variation. Among the four specified types of cyberbullying behaviors, verbal violence was regarded as the most commonly reported behaviors in both perpetration and victimization rates, with a wide range of prevalence, ranging from 5 to 18%. Fewer studies reported the prevalence data for visual violence and group violence. Studies also showed that the prevalence of impersonation and account forgery were within a comparatively small scale. Specific results were as follows.

##### Verbal Violence

A total of 13 studies reported verbal violence prevalence data (15, 26, 34, 37–39, 42, 43, 47, 48, 50, 51). Ten studies reported the prevalence of verbal violence victimization ranging from 2.8 to 47.5%, while seven studies claimed perpetration prevalence ranging from 1.5 to 31.8%. Malaysia reported the highest prevalence of verbal violence victimization (47.5%) (37), followed by China (32%) (43). China reported that the prevalence of verbal violence victimization ranged from 5.1 to 32% (15, 43). Israel reported that the prevalence of verbal violence victimization ranged from 3.4 to 18% (26, 38, 42). For perpetration rate, Malaysia reported the highest level at 31.8% (37), while a study for Spain reported the lowest, ranging from 3.2 to 6.4% (51).

##### Group Violence

The prevalence of group violence victimization was explored within 4 studies and ranged from 5 to 17.8% (26, 34, 42, 43), while perpetration prevalence was reported in three studies, ranging from 10.1 to 19.07% (34, 43, 47). An Israeli study suggested that 9.8% of respondents had been excluded from the Internet, while 8.9% had been refused entry to a group or team (26). A study in South Korea argued that the perpetration prevalence of group violence was 10.1% (34), while a study in Italy reported that the rate of online group violence against others was 19.07% (47).

##### Visual Violence

The prevalence of visual violence victimization was explored within three studies and ranged from 2.6 to 12.1% (26, 34, 43), while the perpetration prevalence reported in four studies ranged from 1.7 to 6% (34, 43, 47, 48). For victimization prevalence, a South Korean study found that 12.1% of respondents reported that their personal information was leaked online (34). An Israel study reported that the prevalence of outing the picture was 2.6% (26). For perpetration prevalence, a South Korean study found that 1.7% of respondents had reported that they had disclosed someone's personal information online (34). A German study reported that 6% of respondents had written a message (e.g., an email) to somebody using a fake identity (48).

##### Impersonating and Account Forgery

Four studies reported on the victimization prevalence of impersonating and account forgery, ranging from 1.1 to 10% (15, 42, 43), while five studies reported on perpetration prevalence, with the range being from 1.3 to 9.31% (15, 43, 47, 48, 51). In a Spanish study, 10% of respondents reported that their accounts had been infringed by others or that they could not access their account due to stolen passwords. In contrast, 4.5% of respondents reported that they had infringed other people's accounts or stolen passwords, with 2.5% stating that they had forged other people's accounts (51). An Israeli study reported that the prevalence of being impersonated was 7% (42), while in China, a study reported this to be 8.6% (43). Another study from China found that 1.1% of respondents had been impersonated to send dating-for-money messages (15).

##### Other Behaviors

The prevalence of disclosure of privacy, sexual harassment, and cyberstalking were also explored by scholars. Six studies reported the victimization prevalence of other cyberbullying behaviors (13, 15, 34, 37, 42, 43), and four studies reported on perpetration prevalence (34, 37, 43, 48). A study in China found that 1.2% of respondents reported that their privacy had been compromised without permission due to disputes (15). A study from China reported the prevalence of cyberstalking victimization was 11.9% (43), while a Portuguese study reported that this was 62% (13). In terms of perpetration prevalence, a Malaysian study reported 2.7% for sexual harassment (37).

### Risk and Protective Factors of Cyberbullying

In terms of the risk factors associated with cyberbullying among children and adolescents, this comprehensive review highlighted both personal and situational factors. Personal factors referred to age, gender, online behavior, race, health conditions, past experiences of victimization, and impulsiveness, while situational factors consisted of parent-child relationship, interpersonal relationships, and geographical location. In addition, protective factors against cyberbullying included: empathy and emotional intelligence, parent-child relationship, and school climate. Table 2 shows the risk and protective factors for child and adolescent cyberbullying.

**Table 2:** Risk and protective factors of cyberbullying among children and adolescents.

**Level**	**Risk factors**	**Protective factors**
Personal factors (victimization)	Age (15, 26, 33, 38, 52, 53)	Empathy and emotional intelligence (34, 45, 48, 54–57)
	Gender (13, 26, 29, 38, 43, 46, 52, 54, 55, 58, 59)	
	Online behavior (32, 36, 43, 48, 49, 60)	
	Race (29, 52)	
	Health condition (29, 33, 41, 52, 61–66)	
Personal factors (perpetration)	Age (55, 67)	–
	Gender (34, 39, 42, 55, 56, 61, 68–71)	
	Online behavior (49, 55)	
	Past experience of victimization (35, 42, 49, 51, 55)	
	Impulsiveness (55, 72)	
Situational factors	Parent-child relationship (19, 33, 43, 64, 68, 73–77)	Parent-child relationship (31, 45, 46, 50, 55, 68, 71, 73, 74)
	Interpersonal relationship (33, 52, 61, 78)	School climate (33, 44, 61, 79)
	Geographical location (49, 61)	

In terms of the risk factors associated with cyberbullying victimization at the personal level, many studies evidenced that females were more likely to be cyberbullied than males (13, 26, 29, 38, 43, 52, 54, 55, 58). Meanwhile, adolescents with mental health problems (61), such as depression (33, 62), borderline personality disorder (63), eating disorders (41), sleep deprivation (56), and suicidal thoughts and suicide plans (64), were more likely to be associated with cyberbullying victimization. As for Internet usage, researchers agreed that youth victims were probably those that spent more time online than their counterparts (32, 36, 43, 45, 48, 49, 60). For situational risk factors, some studies have proven the relationship between cyberbullying victims and parental abuse, parental neglect, family dysfunction, inadequate monitoring, and parents' inconsistency in mediation, as well as communication issues (33, 64, 68, 73). In terms of geographical location, some studies have reported that youths residing in city locations are more likely to be victims of cyberbullying than their peers from suburban areas (61).

Regarding the risk factors of cyberbullying perpetration at the personal level, it is generally believed that older teenagers, especially those aged over 15 years, are at greater risk of becoming cyberbullying perpetrators (55, 67). When considering prior cyberbullying experiences, evidence showed that individuals who had experienced cyberbullying or face-to-face bullying tended to be aggressors in cyberbullying (35, 42, 49, 51, 55); in addition, the relationship between impulsiveness and cyberbullying perpetration was also explored by several pioneering scholars (55, 72, 80). The situational factors highlight the role of parents and teachers in cyberbullying experiences. For example, over-control and authoritarian parenting styles, as well as inharmonious teacher-student relationships (61) are perceived to lead to cyberbullying behaviors (74, 75). In terms of differences in geographical locations, students residing in cities have a higher rate of online harassment than students living in more rural locations (49).

In terms of the protective factors in child and adolescent cyberbullying, scholars have focused on youths who have limited experiences of cyberbullying. At the personal level, high emotional intelligence, an ability for emotional self-control and empathy, such as cognitive empathy ability (44, 55), were associated with lower rates of cyberbullying (57). At the situational level, a parent's role is seen as critical. For example, intimate parent-child relationships (46) and open active communication (19) were demonstrated to be related to lower experiences of cyberbullying and perpetration. Some scholars argued that parental supervision and monitoring of children's online activities can reduce their tendency to participate in some negative activities associated with cyberbullying (31, 46, 73). They further claimed that an authoritative parental style protects youths against cyberbullying (43). Conversely, another string of studies evidenced that parents' supervision of Internet usage was meaningless (45). In addition to conflicting roles of parental supervision, researchers have also looked into the role of schools, and posited that positive school climates contribute to less cyberbullying experiences (61, 79).

Some risk factors may be protective factors under another condition. Some studies suggest that parental aggressive communication is related to severe cyberbullying victims, while open communication is a potential protective factor (19). Parental neglect, parental abuse, parental inconsistency in supervision of adolescents' online behavior, and family dysfunction are related to the direct or indirect harm of cyberbullying (33, 68). Parental participation, a good parental-children relationship, communication and dialogue can enhance children's school adaptability and prevent cyberbullying behaviors (31, 74). When parental monitoring reaches a balance between control and openness, it could become a protective factor against cyberbullying, and it could be a risk factor, if parental monitoring is too low or over-controlled (47).

Despite frequent discussion about the risk factors associated with cyberbullying among children and adolescents, some are still deemed controversial factors, such as age, race, gender, and the frequency of suffering on the internet. For cyberbullying victims, some studies claim that older teenagers are more vulnerable to cyberbullying (15, 38, 52, 53), while other studies found conflicting results (26, 33). As for student race, Alhajji et al. argued that non-white students were less likely to report cyberbullying (29), while Morin et al. observed no significant correlation between race and cyberbullying (52). For cyberbullying perpetration, Alvarez-Garcia found that gender differences may have indirect effects on cyberbullying perpetration (55), while others disagreed (42, 61, 68–70). Specifically, some studies revealed that males were more likely to become cyberbullying perpetrators (34, 39, 56), while Khurana et al. presented an opposite point of view, proposing that females were more likely to attack others (71). In terms of time spent on the Internet, some claimed that students who frequently surf the Internet had a higher chance of becoming perpetrators (49), while others stated that there was no clear and direct association between Internet usage and cyberbullying perpetration (55).

In addition to personal and situational factors, scholars have also explored other specific factors pertaining to cyberbullying risk and protection. For instance, mindfulness and depression were found to be significantly related to cyber perpetration (76), while eating disorder psychopathology in adolescents was associated with cyber victimization (41). For males who were familiar with their victims, such as family members, friends and acquaintances, they were more likely to be cyberstalking perpetrators than females or strangers, while pursuing desired closer relationships (13). In the school context, a lower social likability in class was identified as an indirect factor for cyberbullying (48).

## Discussion

This comprehensive review has established that the prevalence of global childhood and adolescent victimization from cyberbullying ranges from 13.99 to 57.5%, and that the perpetration prevalence ranges from 6.0 to 46.3%. Across the studies included in our research, verbal violence is observed as one of the most common acts of cyberbullying, including verbal offensive responses, insults, mocking, threats, slander, and harassment. The victimization prevalence of verbal violence is reported to be between 5 and 47.5%, and the perpetration prevalence is between 3.2 and 26.1%. Personal factors, such as gender, frequent use of social media platforms, depression, borderline personality disorder, eating disorders, sleep deprivation, and suicidal tendencies, were generally considered to be related to becoming a cyberbullying victim. Personal factors, such as high school students, past experiences, impulse, improperly controlled family education, poor teacher-student relationships, and the urban environment, were considered risk factors for cyberbullying perpetration. Situational factors, including parental abuse and neglect, improper monitoring, communication barriers between parents and children, as well as the urban environment, were also seen to potentially contribute to higher risks of both cyberbullying victimization and perpetration.

### Increasing Prevalence of Global Cyberbullying With Changing Social Media Landscape and Measurement Alterations

This comprehensive review suggests that global cyberbullying rates, in terms of victimization and perpetration, were on the rise during the 5 year period, from 2015 to 2019. For example, in an earlier study conducted by Modecki et al. the average cyberbullying involvement rate was 15% (81). Similar observations were made by Hamm et al. who found that the median rates of youth having experienced bullying or who had bullied others online, was 23 and 15.2%, respectively (82). However, our systematic review summarized global children and adolescents cyberbullying in the last 5 years and revealed an average cyberbullying perpetration rate of 25.03%, ranging from 6.0 to 46.3%, while the average victimization was 33.08%, ranging from 13.99 to 57.5%. The underlying reason for increases may be attributed to the rapid changing landscape of social media and, in recent years, the drastic increase in Internet penetration rates. With the rise in Internet access, youths have greater opportunities to participate in online activities, provided by emerging social media platforms.

Although our review aims to provide a broader picture of cyberbullying, it is well-noted in extant research that difficulties exist in accurately estimating variations in prevalence in different countries (23, 83). Many reasons exist to explain this. The first largely relates poor or unclear definition of the term cyberbullying; this hinders the determination of cyberbullying victimization and perpetration (84). Although traditional bullying behavior is well-defined, the definition cannot directly be applied to the virtual environment due to the complexity in changing online interactions. Without consensus on definitions, measurement and cyberbullying types may vary noticeably (83, 85). Secondly, the estimation of prevalence of cyberbullying is heavily affected by research methods, such as recall period (lifetime, last year, last 6 months, last month, or last week etc.), demographic characteristics of the survey sample (age, gender, race, etc.), perspectives of cyberbullying experiences (victims, perpetrators, or both victim and perpetrator), and instruments (scales, study-specific questions) (23, 84, 86). The variety in research tools and instruments used to assess the prevalence of cyberbullying can cause confusion on this issue (84). Thirdly, variations in economic development, cultural backgrounds, human values, internet penetration rates, and frequency of using social media may lead to different conclusions across countries (87).

### Acknowledging the Conflicting Role of the Identified Risk Factors With More Research Needed to Establish the Causality

Although this review has identified many personal and situational factors associated with cyberbullying, the majority of studies adopted a cross-sectional design and failed to reveal the causality (21). Nevertheless, knowledge on these correlational relationships provide valuable insights for understanding and preventing cyberbullying incidents. In terms of gender differences, females are believed to be at a higher risk of cyberbullying victimization compared to males. Two reasons may help to explain this. First, the preferred violence behaviors between two genders. females prefer indirect harassment, such as the spreading of rumors, while males tend toward direct bullying (e.g., assault) (29) and second, the cultural factors. From the traditional gender perspective, females tended to perceive a greater risk of communicating with others on the Internet, while males were more reluctant to express fear, vulnerability and insecurity when asked about their cyberbullying experiences (46). Females were more intolerant when experiencing cyberstalking and were more likely to report victimization experiences than males (13). Meanwhile, many researchers suggested that females are frequent users of emerging digital communication platforms, which increases their risk of unpleasant interpersonal contact and violence. From the perspective of cultural norms and masculinity, the reporting of cyberbullying is also widely acknowledged (37). For example, in addition, engaging in online activities is also regarded as a critical predictor for cyberbullying victimization. Enabled by the Internet, youths can easily find potential victims and start harassment at any time (49). Participating in online activities directly increases the chance of experiencing cyberbullying victimization and the possibility of becoming a victim (36, 45). As for age, earlier involvement on social media and instant messaging tools may increase the chances of experiencing cyberbullying. For example, in Spain, these tools cannot be used without parental permission before the age of 14 (55). Besides, senior students were more likely to be more impulsive and less sympathetic. They may portray more aggressive and anti-social behaviors (55, 72); hence senior students and students with higher impulsivity were usually more likely to become cyberbullying perpetrators.

Past experiences of victimization and family-related factors are another risk for cyberbullying crime. As for past experiences, one possible explanation is that young people who had experienced online or traditional school bullying may commit cyberbullying using e-mails, instant messages, and text messages for revenge, self-protection, or improving their social status (35, 42, 49, 55). In becoming a cyberbullying perpetrator, the student may feel more powerful and superior, externalizing angry feelings and relieving the feelings of helplessness and sadness produced by past victimization experiences (51). As for family related factors, parenting styles are proven to be highly correlated to cyberbullying. In authoritative families, parents focus on rational behavioral control with clear rules and a high component of supervision and parental warmth, which have beneficial effects on children's lifestyles (43). Conversely, in indulgent families, children's behaviors are not heavily restricted and parents guide and encourage their children to adapt to society. The characteristics of this indulgent style, including parental support, positive communication, low imposition, and emotional expressiveness, possibly contribute to more parent-child trust and less misunderstanding (75). The protective role of warmth/affection and appropriate supervision, which are common features of authoritative or indulgent parenting styles, mitigate youth engagement in cyberbullying. On the contrary, authoritarian and neglectful styles, whether with excessive or insufficient control, are both proven to be risk factors for being a target of cyberbullying (33, 76). In terms of geographical location, although several studies found that children residing in urban areas were more likely to be cyberbullying victims than those living in rural or suburban areas, we cannot draw a quick conclusion here, since whether this difference attributes to macro-level differences, such as community safety or socioeconomic status, or micro-level differences, such as teacher intervention in the classroom, courses provided, teacher-student ratio, is unclear across studies (61). An alternative explanation for this is the higher internet usage rate in urban areas (49).

Regarding health conditions, especially mental health, some scholars believe that young people with health problems are more likely to be identified as victims than people without health problems. They perceive health condition as a risk factor for cyberbullying (61, 63). On the other hand, another group of scholars believe that cyberbullying has an important impact on the mental health of adolescents which can cause psychological distress consequences, such as post-traumatic stress mental disorder, depression, suicidal ideation, and drug abuse (70, 87). It is highly possible that mental health could be risk factors, consequences of cyberbullying or both. Mental health cannot be used as standards, requirements, or decisive responses in cyberbullying research (13).

### The Joint Effort Between Youth, Parents, Schools, and Communities to Form a Cyberbullying-Free Environment

This comprehensive review suggests that protecting children and adolescents from cyberbullying requires joint efforts between individuals, parents, schools, and communities, to form a cyberbullying-free environment. For individuals, young people are expected to improve their digital technology capabilities, especially in the use of social media platforms and instant messaging tools (55). To reduce the number of cyberbullying perpetrators, it is necessary to cultivate emotional self-regulation ability through appropriate emotional management training. Moreover, teachers, counselors, and parents are required to be armed with sufficient knowledge of emotional management and to develop emotional management capabilities and skills. In this way, they can be alert to the aggressive or angry emotions expressed by young people, and help them mediate any negative emotions (45), and avoid further anti-social behaviors (57).

For parents, styles of parenting involving a high level of parental involvement, care and support, are desirable in reducing the possibility of children's engagement in cyberbullying (74, 75). If difficulties are encountered, open communication can contribute to enhancing the sense of security (73). In this vein, parents should be aware of the importance of caring, communicating and supervising their children, and participate actively in their children's lives (71). In order to keep a balance between control and openness (47), parents can engage in unbiased open communication with their children, and reach an agreement on the usage of computers and smart phones (34, 35, 55). Similarly, it is of vital importance to establish a positive communication channel with children (19).

For schools, a higher priority is needed to create a safe and positive campus environment, providing students with learning opportunities and ensuring that every student is treated equally. With a youth-friendly environment, students are able to focus more on their academic performance and develop a strong sense of belonging to the school (79). For countries recognizing collectivist cultural values, such as China and India, emphasizing peer attachment and a sense of collectivism can reduce the risk of cyberbullying perpetration and victimization (78). Besides, schools can cooperate with mental health agencies and neighboring communities to develop preventive programs, such as extracurricular activities and training (44, 53, 62). Specifically, school-based preventive measures against cyberbullying are expected to be sensitive to the characteristics of young people at different ages, and the intersection of race and school diversity (29, 76). It is recommended that school policies that aim to embrace diversity and embody mutual respect among students are created (26). Considering the high prevalence of cyberbullying and a series of serious consequences, it is suggested that intervention against cyberbullying starts from an early stage, at about 10 years old (54). Schools can organize seminars to strengthen communication between teachers and students so that they can better understand the needs of students (61). In addition, schools should encourage cyberbullying victims to seek help and provide students with opportunities to report cyberbullying behaviors, such as creating online anonymous calls.

## Conclusions and Limitations

The comprehensive study has reviewed related research on children and adolescents cyberbullying across different countries and regions, providing a positive understanding of the current situation of cyberbullying. The number of studies on cyberbullying has surged in the last 5 years, especially those related to risk factors and protective factors of cyberbullying. However, research on effective prevention is insufficient and evaluation of policy tools for cyberbullying intervention is a nascent research field. Our comprehensive review concludes with possible strategies for cyberbullying prevention, including personal emotion management, digital ability training, policy applicability, and interpersonal skills. We highlight the important role of parental control in cyberbullying prevention. As for the role of parental control, it depends on whether children believe their parents are capable of adequately supporting them, rather than simply interfering in their lives, restricting their online behavior, and controlling or removing their devices (50). In general, cyberbullying is on the rise, with the effectiveness of interventions to meet this problem still requiring further development and exploration (83).

Considering the overlaps between cyberbullying and traditional offline bullying, future research can explore the unique risk and protective factors that are distinguishable from traditional bullying (86). To further reveal the variations, researchers can compare the outcomes of interventions conducted in cyberbullying and traditional bullying preventions simultaneously, and the same interventions only targeting cyberbullying (88). In addition, cyberbullying also reflects a series of other social issues, such as personal privacy and security, public opinion monitoring, multinational perpetration and group crimes. To address this problem, efforts from multiple disciplines and novel analytical methods in the digital era are required. As the Internet provides enormous opportunities to connect young people from all over the world, cyberbullying perpetrators may come from transnational networks. Hence, cyberbullying of children and adolescents, involving multiple countries, is worth further attention.

Our study has several limitations. First, national representative studies are scarce, while few studies from middle and low income countries were included in our research due to language restrictions. Many of the studies included were conducted in schools, communities, provinces, and cities in high income countries. Meanwhile, our review only focused on victimization and perpetration. Future studies should consider more perspectives, such as bystanders and those with the dual identity of victim/perpetrator, to comprehensively analyze the risk and protective factors of cyberbullying.

## Data Availability Statement

The original contributions presented in the study are included in the article/Supplementary Material, further inquiries can be directed to the corresponding author/s.

## Author Contributions

SH, CZ, RE, and WZ conceived the study and developed the design. WZ analyzed the result and supervised the study. CZ and SH wrote the first draft. All authors contributed to the article and approved the submitted version.

## Conflict of Interest

The authors declare that the research was conducted in the absence of any commercial or financial relationships that could be construed as a potential conflict of interest.
