# Supplementary material for: Cyberbullying Among Adolescents and Children: A Comprehensive Review of the Global Situation, Risk Factors, and Preventive Measures
Source: Front Public Health. 2021 Mar 11;9:634909. doi: 10.3389/fpubh.2021.634909 (PMC8006937; doi:10.3389/fpubh.2021.634909)
Supplement: Supplementary file 1 [file Table_1.docx]

Appendix 1. Risk of bias according to Cochrane criteria

| **Reference** | **Random sequence generation** | **Allocation concealment** | **Blinding of participants and personnel** | **Blinding of outcome assessment** | **Incomplete outcome data** | **Selective reporting** | **Other sources of bias** | **Quality assessment** |
| --- | --- | --- | --- | --- | --- | --- | --- | --- |
| Aizenkot et al. 2019 | unclear | unclear | low risk | unclear | unclear | unclear | low risk | middle |
| Alhajji et al. 2019 | low risk | unclear | low risk | unclear | unclear | unclear | unclear | middle |
| Alvarez-Garcia et al. 2018 | low risk | unclear | low risk | unclear | unclear | unclear | low risk | high |
| Alvarez-Garcia et al. 2019 | low risk | unclear | low risk | unclear | unclear | unclear | low risk | high |
| Baldry et al. 2019 | unclear | unclear | low risk | unclear | unclear | unclear | low risk | middle |
| Baraldsnes, 2015 | low risk | low risk | unclear | unclear | unclear | unclear | unclear | middle |
| Beran, 2015 | low risk | low risk | unclear | unclear | low risk | unclear | unclear | high |
| Brighi, 2019 | low risk | unclear | low risk | unclear | unclear | unclear | low risk | high |
| Buelga, 2015 | unclear | unclear | low risk | unclear | unclear | unclear | low risk | middle |
| Carmen Martinez-Monteagudo, 2019 | low risk | unclear | low risk | unclear | unclear | unclear | low risk | high |
| Cénat, 2018 | unclear | low risk | unclear | unclear | unclear | unclear | low risk | middle |
| Dilmac, 2016 | low risk | low risk | unclear | unclear | unclear | unclear | unclear | middle |
| Festl, 2016 | low risk | low risk | low risk | unclear | unclear | unclear | low risk | high |
| Garaigordobil, 2015 | low risk | unclear | unclear | unclear | unclear | unclear | unclear | middle |
| Grinshteyn, 2017 | low risk | unclear | unclear | unclear | low risk | unclear | low risk | high |
| Ho, 2017 | low risk | unclear | unclear | unclear | high risk (The response rates were 26.7% for primary schools and 33.3% for secondary schools and 33.3% for secondary schools) | unclear | low risk | low |
| Hoareau, 2019 | unclear | unclear | low risk | unclear | unclear | unclear | unclear | middle |
| Holfeld, 2017 | low risk | unclear | low risk | unclear | unclear | unclear | low risk | high |
| Holt, 2016 | low risk | low risk | low risk | unclear | low risk | unclear | unclear | high |
| Hong, 2018 | low risk | unclear | unclear | unclear | unclear | unclear | low risk | middle |
| Horzum, 2019 | low risk | unclear | low risk | unclear | unclear | unclear | unclear | middle |
| Huang, 2019 | low risk | unclear | low risk | unclear | unclear | unclear | low risk | high |
| Iranzo, 2019 | low risk | unclear | low risk | unclear | unclear | unclear | low risk | high |
| Katz, 2019 | unclear | unclear | unclear | unclear | unclear | unclear | low risk | middle |
| Khurana, 2015 | low risk | unclear | unclear | unclear | unclear | unclear | low risk | middle |
| Kim, 2018 | low risk | low risk | low risk | unclear | unclear | unclear | low risk | high |
| Landoll, 2015 | Unclear | unclear | high risk (Anonymity was not mentioned, and adolescents were supervised by research assistants.) | unclear | unclear | unclear | low risk | low |
| Larranaga, 2016 | low risk | unclear | low risk | unclear | unclear | unclear | low risk | high |
| Lee, 2017a | low risk | low risk | unclear | unclear | unclear | unclear | low risk | high |
| Lee, 2017b | Unclear | low risk | low risk | unclear | low risk | unclear | unclear | high |
| Lin, 2016 | high risk (This study used non identifiable secondary data from a school-based health surveillance from only one elementary school in Taoyuan, Taiwan. The sampling method was not mentioned.) | unclear | unclear | unclear | unclear | unclear | unclear | low |
| Marco, 2018 | high risk (participants in this study were from several cities in Spain. The sampling method was not mentioned.) | unclear | low risk | unclear | unclear | unclear | low risk | low |
| Marret, 2017 | low risk | unclear | low risk | unclear | unclear | unclear | low risk | high |
| Martínez, 2019 | low risk | unclear | low risk | unclear | unclear | unclear | low risk | high |
| Martinez-Ferrer, 2019 | low risk | unclear | unclear | unclear | unclear | unclear | low risk | middle |
| McQuillan, 2016 | low risk | unclear | low risk | unclear | unclear | unclear | unclear | middle |
| Mesch, 2018 | unclear | unclear | unclear | unclear | unclear | unclear | low risk | middle |
| Moreno–Ruiz, 2019 | low risk | unclear | low risk | unclear | low risk | unclear | low risk | high |
| Morin, 2018 | unclear | low risk | unclear | unclear | low risk | unclear | low risk | high |
| Navarro, 2018 | unclear | unclear | low risk | unclear | unclear | unclear | low risk | middle |
| Olenik-Shemesh, 2017 | unclear | unclear | low risk | unclear | low risk | unclear | unclear | middle |
| Olumide, 2016 | low risk | low risk | low risk | unclear | unclear | unclear | unclear | high |
| Pereira, 2016 | low risk | unclear | unclear | unclear | low risk | unclear | low risk | high |
| Pieschl, 2017 | Unclear | unclear | low risk | unclear | low risk | unclear | low risk | high |
| Chen, 2018 | low risk | unclear | unclear | unclear | unclear | unclear | low risk | middle |
| Rao, 2019 | low risk | low risk | low risk | unclear | low risk | unclear | low risk | high |
| Razjouyan, 2018 | low risk | unclear | low risk | unclear | unclear | unclear | unclear | middle |
| Reed, 2018 | Unclear | low risk | low risk | unclear | unclear | unclear | low risk | high |
| Rose, 2015 | Unclear | unclear | low risk | unclear | unclear | unclear | low risk | middle |
| Sam, 2017 | low risk | unclear | low risk | unclear | unclear | unclear | unclear | middle |
| Sarina, 2018 | low risk | unclear | unclear | unclear | unclear | unclear | unclear | middle |
| Sari, 2016a | Unclear | unclear | unclear | unclear | unclear | unclear | low risk | middle |
| Sari, 2016b | Unclear | unclear | unclear | unclear | unclear | unclear | low risk | middle |
| Sasson, 2017 | low risk | low risk | low risk | unclear | unclear | unclear | unclear | high |
| Simsek, 2019 | low risk | unclear | unclear | unclear | unclear | unclear | unclear | middle |
| Stockdale, 2015 | Unclear | unclear | unclear | unclear | unclear | unclear | low risk | middle |
| Stoll, 2015 | Unclear | unclear | unclear | unclear | unclear | unclear | low risk | middle |
| Tesler, 2019 | low risk | unclear | unclear | unclear | unclear | unclear | unclear | middle |
| Wang, 2016 | high risk (Participants in this study were from only one middle school in Anhui, China. The sampling method was not mentioned.) | unclear | unclear | unclear | unclear | unclear | low risk | low |
| Wright, 2017 | unclear | unclear | unclear | unclear | unclear | unclear | unclear | middle |
| Wright, 2015 | unclear | unclear | unclear | unclear | unclear | unclear | low risk | middle |
| You, 2016 | low risk | unclear | unclear | unclear | low risk | unclear | low risk | high |
| Yuan, 2019 | unclear | unclear | unclear | unclear | low risk | unclear | low risk | middle |
