# Supplementary material for: Cyberbullying Among Adolescents and Children: A Comprehensive Review of the Global Situation, Risk Factors, and Preventive Measures
Source: Front Public Health. 2021 Mar 11;9:634909. doi: 10.3389/fpubh.2021.634909 (PMC8006937; doi:10.3389/fpubh.2021.634909)
Supplement: Supplementary file 2 [file Table_2.docx]

Appendix 2. Characteristics of included studies

| **Author, year** | **Study type** | **Online or face-to-face** | **Recall Period** | **Measures of cyberbullying** | **Self-labeling or behavior experience method** | **Definition of cyberbullying** |
| --- | --- | --- | --- | --- | --- | --- |
| Aizenkot et al. 2019 | Cross-sectional study | Survey face-to-face | In the last 8 weeks | Questionnaire about cyberbullying victimization among WhatsApp classmate groups.  A 10-item questionnaire about cyberbullying victimization or about the victim’s reaction to cyberbullying in these groups. | Behavior experience method | No definition provided |
| Alhajji et al. 2019 | Cross-sectional study | \ | In the last 12 months | The original 2015 YRBS codebook, https://www.cdc.gov/healthyyouth/data/yrbs/. Published in 2016. Accessed July 25, 2019. | Self-labeling | No definition provided |
| Alvarez-Garcia et al. 2018 | Cross-sectional and correlational research | Survey; face-to-face | Polytomous items: such as from 0, never to 3, always | An ad-hoc questionnaire was used to collect information about age, sex, and the grade year of respondents, as well as information referring to the availability and frequency of use of communication technologies; the “behavioral control” “affection and communication” factor from the Dimensions of Parenting Style Questionnaire, proposed by Álvarez-García, four items, five options García, Barreiro-Collazo, Dobarro, and Antúnez (2016); the “rules for Internet use” “monitoring Internet use” factors from the Parental Control on Internet Usage Questionnaire by Álvarez-García, García, Cueli, and Núñez (2017); four items, 4 options The degree of the respondents’ impulsivity was assessed using a self-reported scale, five items, four options; five items, four options; six items, a Likert-type list scale four options (from 0, never to 3, always), dichotomous; the Cyber Victimization Questionnaire for Adolescents ;19 items that refer to five types of cyber-victimization; self-reported scale Cyber-Aggression Questionnaire for Adolescents, both victimization and aggression. | Behavior experience method | No definition provided |
| Alvarez-Garcia et al. 2019 | Cross-sectional study | Survey; face-to-face | Polytomous items: such as from 1 = completely false to 4 = completely true | Parental Control for Adolescent Internet Use Questionnaire;  High-Risk Internet Behaviors Questionnaire;  Impulsivity Scale ;  Cyber Victimization Questionnaire for Adolescents | Behavior experience method | No definition provided |
| Baldry et al. 2019 | Cross-sectional study | Survey; face-to-face | In the last 6 months | The Italian translation of the Students’ Needs Assessment Survey;  Each behavior was measured on a 3-point scale: “no” (scored 0), “yes, 1 to 4 times” (scored 1) and “yes, 5 or more times” (scored 2) | Behavior experience method | Cyberbullying is an aggressive and intentional act carried out by a group or an individual using electronic forms of contact, repeatedly over time against a victim who cannot easily defend himself/herself |
| Baraldsnes, 2015 | Cross-sectional study | \ | 5-points scale (Never, 1-3 times a month, 1-2 times a week, 3-5 times a week, and every day) | Respondents (both pupils and teachers) were asked to fill out a questionnaire of 12 items, consisting of three parts: an introduction, a main part, and demographic questions, together with a definition of cyberbullying; 5-points scale (Never, 1-3 times a month, 1-2 times a week, 3-5 times a week, and every day). | Self-labeling | Patchin’s and  Hinduja’s definition of cyberbullying: “an act of doing intentional and recurrent harm to another person using electronic tools. One pupil or group of pupils can bully other pupil via mobile phones, in the form of calls, text messages and picture/video clips sent to others or uploaded onto a website; and via the Internet, in the form of abusive messages posted online a such as email, chat-room, instant messaging or websites”. |
| Beran, 2015 | Cross-sectional study | Survey online | In the last month | The Cronbach’s alphas of the seven types of victimization and seven types of perpetration items. The subsequent section included eight scales that examine the cognitive, psychological, and behavioral impact of bullying. The Relationships subscale measured children’s reports of positive interactions among peers (six items); Anger measured angry feelings and actions (six items); Anxiety measured nervous thoughts (seven items); Self-Esteem measured self-worth (three items); Risk measured involvement in criminal behaviors (five items); Physical Injury measured physical harm sustained from victimization (three items); Drug Use measured drug consumption (three items); and Eating Problems measured problematic eating behaviors (four items). All 37 items were rated on a five-point frequency scale: 1 = never, 2 = only once or twice, 3 = sometimes, 4 = about once a week, and 5 = several times a week. | Self-labeling | There are lots of ways to hurt someone. A person who bullies wants to hurt  the other person. A person who bullies does it because they can. They may be  older, stronger, bigger, or have other students on their side. There are different kinds of bullying: 1. physical, such as, hitting, or spitting; 2. verbal, such as, name-calling, or mocking; 3. social, such as, leaving someone out, or gossiping. 4. electronic, such as, Facebook, or email; 5. racial, such as, saying hurtful  things about someone whose skin is a different color; 6. sexual, such as, grabbing, or saying something sexual; and 7. sexual preference, such as, teasing someone for being gay whether they are or not. |
| Brighi, 2019 | Cross-sectional study | Online anonymous self-reported questionnaire,  administered during ICT classes. | Lifetime | The Italian version of the cyberbullying scale from the European Cyberbullying Intervention Project Questionnaire. | Behavior experience method | No definition provided |
| Buelga, 2015 | Cross-sectional study | Survey; face-to-face | In the last 12 months | Cyberbullying Aggressive Behaviors Scale (CABS), comprising ten items taken from the CYBVIC scale and adapted to the specific role of perpetrators of behaviors; a five-point Likert-type scale. | Behavior experience method | No definition provided |
| Carmen Martinez-Monteagudo, 2019 | Cross-sectional study | Individualized interviews | In the last 12 months | The Screening for Peer Bullying. The questionnaire contains 45 questions to be responded to on a 4-point Likert scale, ranging from 0 (never) to 3 (always).  A Spanish adaptation of the Aggression Questionnaire consists of 29 items, answered using a 5-point Likert scale. | Behavior experience method | No definition provided |
| Cénat, 2018 | Longitudinal design | The two first waves of data were collected in schools, while the third wave participants were invited to complete the survey online via a secured server | Measuring cyberbullying victimization occurring in the last 12 months | Three waves conducted six months apart; A specific question measuring cyberbullying victimization;  the Kessler Psychological Distress encompasses 10 items rated on a 5-point-scale ranging from 1(never) to 5 (always) with a score ranging from 10 to 50 (α = 0.90). | Behavior experience method | This item completely covers the definition of the cyberbullying theoretical construct: (1) intentionality of aggressive behavior, and (2) occurs through electronic technologies for cyberbullying. |
| Dilmac,2016 | Cross-sectional study | At a single session | Lifetime | “Humane Values Scale,” the “Cyberbullying Scale” and the “Cyberbullying Sensibility’’ scale. | Self-labeling | definition provided |
| Festl，2016 | Longitudinal design | During lessons in school | In the last 6 months | Different behavioral criteria following the classification of Vandebosch & van Cleemput (2009). | Behavior experience method | definition provided |
| Garaigordobil, 2015 | Cross-sectional study | \ | In the last year | The Cyberbullying Test, which assesses 15 cyberbullying behaviors. | Behavior experience method | No definition provided |
| Grinshteyn, 2017 | Cross-sectional study | A paper-and-pencil questionnaire | In the last 12 months | The YRBS survey instrument. | Self-labeling | No definition provided |
| Ho, 2017 | Cross-sectional study | A self-administered paper-and-pencil survey | In the last 12 months | A 3-item scale adapted from Ybarra, Diener-West, and Leaf (2007). | Behavior experience method | No definition provided |
| Hoareau, 2019 | Cross-sectional study | Survey; face-to-face | \ | A French version of the Electronic Bullying Questionnaire created by Kowalski and Limber (2007) for 11–15 year-olds; The scale has seven items concerning the frequency with which adolescents had ever perpetrated the different cyberbullying behaviors, and whether via websites or cell phones (e.g., I bullied through an email message). The items are rated on a five-point Likert type scale ranging from 1 (never) to 5 (more than once a week). | Behavior experience method | No definition provided |
| Holfeld, 2017 | Cross-lagged models | Survey; face-to-face | In the last 30 days | Four-items from the Cyber Bullying Behaviors and Victimization Experiences for Elementary School Children Scale; a five-point Likert scale ranging  from 0 (never) to 4 (every day). | Behavior experience method | No definition provided |
| Holt, 2016 | Cross-sectional study | Survey; face-to-face | In the last year | A self-reported questionnaire; a 7-item Likert-type scale question with pictorial responses. | Behavior experience method | Any treatment that a person receives that is deliberate, intended to hurt, unjustified, repeated, and carried out by a more powerful person or group, in keeping with the larger research literature on this issue. |
| Hong, 2018 | Cross-sectional study | Not mentioned | In the last 12 months | Cyber Bullying Questionnaire, developed by the Korean National Youth Policy Institute. | Behavior experience method | No definition provided |
| Horzum, 2019 | Cross-sectional study | Paper-pencil method | \ | A cyberbully/victim scale, developed by Ayas and Horzum (2010), for primary school students and adapted for adolescents by Horzum and Ayas (2011) was used to determine the level of cyberbully/victim. There were 17 items in the scale adapted for adolescents. | Not mentioned | No definition provided |
| Huang, 2019 | Cross-sectional study | Paper-and-pencil questionnaires | In the last six months | A CB was developed in online games scale (CBOGS). | Behavior experience method | No definition provided |
| Iranzo, 2019 | Cross-sectional study | During school hours | In the last 12 months | This scale consists of 18 items rated on a 4-point Likert-type scale ranging from 1 (never) to 4 (always). | Behavior experience method | No definition provided |
| Katz, 2019 | Cross-sectional study | \ | In the previous few months | Items from the scales by Walrave and Heirman (2011), and the Cyberbullying Inventory (CBI), Twelve items represent cyberbullying and 12 represent cyber victimization. | Behavior experience method | No definition provided |
| Khurana, 2015 | Cross-sectional study | Online | In the last 12 months | Self-designed questionnaire. | Behavior experience method | No definition provided |
| Kim, 2018 | Cross-sectional study | \ | In the last 6 months | A 5-point scale; items came from the Ontario Ministry of Education’s Safe Schools Survey. | Behavior experience method | A definition of bullying was provided in the survey and included three characteristics of bullying, suggested by Olweus (Olweus 1993, 1994): (1) power imbalance; (2) repetitiveness; and (3) intention to harm. |
| Landoll, 2015 | Prospective study | During class | In the last 2 months | The Cyber-Peer Experiences Questionnaire (C-PEQ); 10 items from the Social Networking e Peer Experiences Questionnaire were modified to make them more general; four new items were added to the C-PEQ. | Behavior experience method | No definition provided |
| Larranaga, 2016 | Cross-sectional study | Both | In the last 3 months | The Spanish Cyberbullying Questionnaire (CBQ-V);  A 10-item self-reported measure. | Behavior experience method | No definition provided |
| Lee, 2017a | Cross-sectional study | \ | In the last 3 months | Developed a cyberbullying scale with eight items. | Behavior experience method | An act of insulting or harassing others by using digital services, such as Kakaotalk and Facebook |
| Lee, 2017b | Cross-sectional study | At computer workstations | Lifetime; In the last 30 days | Cyberbullying offences were measured as a first-order latent factor model, including nine items; nine forms of cyberbullying were combined into a binary variable. | Behavior experience method | Cyberbullying is when  someone repeatedly harasses, mistreats, or makes fun of another person online or while using cell phones or other electronic devices |
| Lin, 2016 | Cross-sectional study | \ | \ | Cyber victimization was measured using the eight-item General Online Victimization Subscale, answered on a 6-point Likert-type scale. Cyberbullying perpetration was assessed using a 10-item scale developed by the Criminal Investigation Bureau (2008) of Taiwan on a 3-point Likert-type scale. | Behavior experience method | No definition provided |
| Marco, 2018 | Cross-sectional study | During their normal school day | In the last 2 months | The Spanish version of the ECIPQ, composed of a 22-item Likert-type scale with five response options for frequency (from never = 0 to more than once a week = 3). | Behavior experience method | No definition provided |
| Marret, 2017 | Cross-sectional study | Paper and pencil questionnaire; In class in a single  session | In the last 12 months; Lifetime. | The Growing Up with Media Survey and the Youth Internet Safety Survey; International Society for the Prevention of Child Abuse and Neglect (ISPCAN) Child Abuse Screening tool. | Behavior experience method | No definition provided |
| Martínez, 2019 | Cross-sectional study | \ | 回答频率 | 10 items of the Electronic Bullying Questionnaire. | Behavior experience method | No definition provided |
| Martinez-Ferrer, 2019 | Cross-sectional study | \ | In the last 12 months | The Cyber-Aggression Scale consisting of 24 items that measure involvement in violent behaviors through the use of digital media. | Behavior experience method | No definition provided |
| McQuillan, 2016 | Cross-sectional study | A paper survey | \ | An adapted version of the Cyberbullying and Online Aggression Survey. | Behavior experience method | No definition provided |
| Mesch, 2018 | Cross-sectional study | Telephone  interviews via landlines and cell phones; Face to face. | In the last 12 months | A single item that asked teenagers to indicate if, in the last 12 months, they had been bullied online through email, IM, or on a SNS. | Self-labeling | No definition provided |
| Moreno–Ruiz, 2019 | Cross-sectional study | During a regular class period | In the last 12 months | The Adolescent Victimization through Mobile Phone and Internet Scale, consisting of 18 Likert-type items with responses ranging from 1 (never) to4 (always). The Cyberbullying Scale, consists of 10 Likert-type items with a response range of 1 (never) to 5 (very often). | Behavior experience method | No definition provided |
| Morin, 2018 | Cross-sectional study | Web-based survey | In the last 3 months | Self-designed questionnaire. | Self-labeling | Cyberbullying involves posting or sending electronic messages (text, pictures, video) that result in a person feeling hurt, humiliated, or like a victim. |
| Navarro,2018 | Cross-sectional study | Face-to-face | 回答频率 | The scale used was the Spanish Cyberbullying Questionnaire;10-item. | Behavior experience method | No definition provided |
| Olenik-Shemesh, 2017 | Cross-sectional study | In their classes over a period of an hour | In the last year | The Student Survey Questionnaire of Cyberbullying. | Behavior experience method | No definition provided |
| Olumide, 2016 | Cross-sectional study | Using laptop computers | In the last 3 months | Self-designed questionnaire. | Behavior experience method | The paper explained harassment through electronic media was a situation in which a person or a group of people: said mean and/or hurtful things; made fun of him or her; called him or her mean and hurtful names; completely ignored or excluded him or her from a group; left him or her out of things on purpose; told lies or spread false rumors about him or her; sent mean notes; tried to make other students dislike him or her; and other similar hurtful things. Typically, this was carried out by text messaging, pictures/photos or video clips, phone calls, email, chat rooms, and websites. Preferred to use the term ‘harassment’ as opposed to ‘bullying’. |
| Pereira, 2016 | Cross-sectional study | online | During their lifetime | Cyber-Harassment Assessment Scale. | Behavior experience method | No definition provided |
| Pieschl, 2017 | Cross-sectional study | Electronically via Unipark | In the last 2 months | Self-designed questionnaire. | Behavior experience method | No definition provided |
| Chen, 2018 | Cross-sectional study | In a private room at school under the instruction of trained interviewers. | In the last year | The Chinese version of the 34-item Juvenile Victimization Questionnaire (JVQ); employed the Relational Aggression Scale (RAS) | Behavior experience method | No definition provided |
| Rao, 2019 | cross-sectional study | Paper-and-pencil survey; after class by the principal investigator in the classroom | In the last 6 months | Self-designed questionnaire. | Behavior experience method | Seven forms of cyberbullying were defined: flaming, harassment, exclusion, denigration, outing, cyberstalking, and internet fraud. |
| Razjouyan, 2018 | Cross-sectional study | \ | \ | Self-designed. A questionnaire with five major scales: Intrapersonal, Interpersonal, Stress Management, Adaptability, and General Mood. | Not mentioned | No definition provided |
| Reed, 2018 | Cross-sectional study | Computers in a school library media center during one  day of class time with the principal investigator present | \ | A 36-item measure adapted  from a previous DDA. | Behavior experience method | Digital Direct Aggression Perpetration (eight items, α = .81) involved intentional digital behaviors meant to hurt, humiliate, or threaten a dating partner using social media or a mobile phone. |
| Rose, 2015 | Cross-sectional study | \ | In the last year | The six-item Online Bullying and Harassment subscale from the Online Victimization Scale. | Behavior experience method | No definition provided |
| Sam, 2017 | Cross-sectional study | Web-based survey | In the last year | An online harassment scale. This behavioral-based measure consists of 6-items with two subscales: cyberbullying (3 items) and cybervictimization (3 items). | Behavior experience method | No definition provided |
| Sarina, 2018 | Cross-sectional study | In a library, a counseling room, a multipurpose hall or a school canteen for  the sessions | In the last 9 months | Constructed from the previous work of Finkelhor, Mitchell and Wolak, (2001),  and Livingstone et al. (2011) scales; self-administered survey form comprised of 10 items. | Behavior experience method | No definition provided |
| Sari, 2016a | Cross-sectional study | In a class environment, | In the last 6 months | The Cyber Bullying Inventory, revised by Topçu and Baker (2010). | Behavior experience method | No definition provided |
| Sari, 2016b | Cross-sectional study | \ | In the last 6 months | Revised Cyber Bullying Inventory (RCBI) | Behavior experience method | No definition provided |
| Sasson, 2017 | Cross-sectional study | Online survey | In the last year | Self-designed questionnaire. | Behavior experience method | A short paragraph presenting a general definition of bullying behavior adapted from the research questionnaire of the European Union (EU) Kids Online (2010) |
| Simsek, 2019 | Cross-sectional study | \ | \ | The Cyber Victimization and Cyberbullying Scale was developed by Çetin, Yaman, and Peker (2011). It consists of 22 items and two parallel forms of cyberbullying and cyber victimization. | Behavior experience method | No definition provided |
| Stockdale, 2015 | Longitudinal follow-up study | \ |  | Self-designed questionnaire. | Behavior experience method | No definition provided |
| Stoll, 2015 | Cross-sectional study | \ | In the last 30 days | Self-designed questionnaire. | Behavior experience method | No definition provided |
| Tesler, 2019 | Cross-sectional study | \ | In the last 2 months | Self-designed questionnaire. | Behavior experience method | No definition provided |
| Wang, 2016 | Cross-sectional study | \ | \ | Developed by Wright (2014) consists 9  items | Behavior experience method | No definition provided |
| Wright, 2017 | Longitudinal study | In the classroom | \ | Self-designed: four subscales: cyberbullying perpetration, cybervictimization, cyber-trolling perpetration, and cyber-trolling victimization. | Behavior experience method | No definition provided |
| Wright, 2015 | Cross-sectional study | \ | \ | Cyber aggression involvement included 18 items used to assess how often adolescents perpetrated cyber aggression and/or were victimized by cyber aggression. | Behavior experience method | No definition provided |
| You, 2016 | Longitudinal study | \ | \ | The Cyber Bullying Inventory revised by the National Youth Policy Institute to adapt to Korean adolescents' cyberbullying | Behavior experience method | No definition provided |
| Yuan, 2019 | Longitudinal study | \ | In the last 6 months | The Chinese version of Revised Cyber Bullying Inventory Cyberbullying Subscale. | Behavior experience method | No definition provided |
