# Supplementary material for: Cyberbullying Among Adolescents and Children: A Comprehensive Review of the Global Situation, Risk Factors, and Preventive Measures
Source: Front Public Health. 2021 Mar 11;9:634909. doi: 10.3389/fpubh.2021.634909 (PMC8006937; doi:10.3389/fpubh.2021.634909)
Supplement: Supplementary file 3 [file Table_3.docx]

Appendix 3. Cyberbullying Prevalence Estimates, Risk Factors, and Characteristics of Existing Studies

***p < 0.001.

**p < 0.01.

*p < 0.05.

| **Author, year** | **Countries** | **Sample characteristics** | | |  | **The prevalence** | | **Risk factors, OR [95%CI]** |
| --- | --- | --- | --- | --- | --- | --- | --- | --- |
|  |  | **Sampling procedures** | **Age or grade** | **Size** | **%Female** | **Victimization** | **Perpetration** |  |
| Aizenkot et al. 2019 | Israel | 194 classes in 28 public schools, elementary schools—fourth to sixth grades (n = 2,940, 65%)—middle schools—seventh to ninth grades (n = 1,326, 30%)—and a high school—10th to 12th grades (n = 211, 5%). | Elementary, middle, and high school | 4477 | 52.00% | 1. Verbal: 17.60%, Offensive responses：10.1%,   Insult：9.20%,  Mocking:6.40%,  Threats:3.40%,  Curses:6.60%,   1. Outing of pictures：2.60%, 2. Selective membership: 15.1%,   Exclusion from groups：9.80%,  Denied acceptance to  WhatsApp groups:8.90%,  Shunning: 1.5%,   1. Participation avoidance:10.80%, | \ | - Cyberbullying Victimization (CV):   School grade level, OR=2.14, [95%CI: 1.89-2.43] ***  Gender, OR=1.38, [95%CI: 1.22-1.59]*** |
| Alhajji et al. 2019 | United States | the nationally representative YRBS data collected biennially by the Centers for Disease Control and Prevention (CDC) with students in grades 9 to 12 in national public and private schools. A weight is applied to each student record to adjust for nonresponse and the distribution of students by grade, sex, and race in each jurisdiction. The primary sampling units consist of counties, groups of smaller adjacent counties, or subareas of large counties. There was a total of 53 primary sampling units. | Grades 9-12 | 15465 | 48.70% | 15.5% [95%CI: 14.5-16.6] | \ | - Cyberbullying Victimization (CV):   Sex (female), OR=2.4 [1.9-2.9]***  Race (non-white), OR=0.5 [0.4-0.7]**  Depressive symptoms, OR=2.7 [2.1-3.4] ***  Suicidal ideation, OR=1.6 [1.4-1.9] ***  Suicide planning, OR=1.6 [1.2-2.0] ***  Physical fight, OR=1.7 [1.4-2.2] ***  Carried weapon, OR=1.3 [1.0-1.5] ** |
| Alvarez-Garcia et al. 2018 | Spain | Stratified random and cluster sampling from the total number of students in Compulsory Secondary Education (CSE) in Asturias;  19 schools selected | 12-18 years | 3059 | 48.40% | \ | \ | - Cyber-aggressor   Impulsivity, OR=1.19[1.01-1.42]*  Empathy, OR=0.78 (0.65-0.93)**  School aggression, OR=1.91 (1.60-2.27)***  Cyberbullying victim experience, OR=1.57 (1.42-1.75)*** |
| Alvarez-Garcia et al. 2019 | Spain | 20 schools were selected by a combination  of stratified and cluster random sampling from all Compulsory  Secondary Education schools | 11-18 years | 3360 | 48.30% | \ | \ | - Cyberbullying Victimization (CV):   Restriction,  Supervision,  Impulsiveness,  High-risk behavior |
| Baldry et al. 2019 | Italy | In different geographical areas in the Northern and Southern parts of Italy | 13-20 years | 4390 | 55.10% | 30.10%（Male） 32.50%（Female） | 36.50%（Male） 22.90%（Female） | - Cyberbullying Victimization (CV):   Gender,  Age, boys’ OR=1.05 [95%CI: 0.94-1.17], girls’ OR=1.51** [95%CI: 1.04-1.27]  Number of hours on Internet, boys’ OR=1.13* [95%CI: 1.02-1.24], girls’ OR=1.21*** [95%CI: 1.10-1.34]  Parental education on Internet  use, boys’ OR=1.16 [95%CI: 0.93-1.44], girls’ OR=1.01 [95%CI: 0.82-1.24]  Parental control of online activities, boys’ OR=0.96 [95%CI: 0.78-1.16], girls’ OR=1.02 [95%CI: 0.86-1.20]  Parental social network supervision, boys’ OR=1.10 [95%CI: 0.93-1.32], girls’ OR=1.37*** [95%CI: 1.17-1.60] |
| Baraldsnes, 2015 | Lithuanian | 287 schools in 10 different counties in Lithuania. Two-stage selection was applied: in the first stage 10 counties in Lithuania were selected by random sampling. | Grades 5-12 | 2064 | 55.96% | \ | \ | - the Experience of Cyberbullying   Age, by using the  Internet,, by using the mobile phone  Gender, by using the  Internet; by using the mobile phone, |
| Beran et al. 2015 | Canada | The sample was stratified based on age to obtain a representative sample of children ages 10 to 17 years from all 10 provinces of Canada. | 10-17 years | 1001 | \ | 13.99% | 7.99% | - Cyber-Perpetration   Gender |
| Brighi et al. 2019 | Italy | A two-stage, non-probabilistic sampling method. approximate a representative sample of the students in the Emilia Romagna region of Italy | Secondary schools | 3602 | 44.00% | \ | Said unpleasant things:21.69% and 26.91%,  Insulted:16.38%,  Group violence:19.07%,  Visual violence:5.82%  Violated others account:9.31%,  Created a fake account:8.52% | - Cyberbullying   Parental monitoring  emotional symptoms  online time |
| Buelga et al. 2015 | Spain | 9 schools from Valencia, Alicante and Castellon in Spain | 12-17 years | 1415 | 47.00% | \ | 32% | - Age   I sent or manipulated videos or images of someone without their permission,  Exclusion  I pretended I was another person to say or do bad things on the telephone or online   - Persecution behaviours   Gender(boys) |
| Carmen Martinez-Monteagudo et al. 2019 | Spain | Spanish high school and baccalaureate students | 12-18 years | 1102 | 54.72% | \ | \ | - Victim   Physical Aggression, OR=1.03 [95%CI: 1.00–1.06]*  Anger, OR=1.06 [95%CI: 1.02–1.08]**  Emotional intelligence, OR=0.94 [95%CI: 0.92–0.97]   - Aggressor   Physical Aggression, OR=1.05 [95%CI: 1.02–1.08]***  Anger, OR=1.08 [95%CI: 1.04–1.10]***  Emotional intelligence, OR=0.94 [95%CI: 0.92–0.97] |
| Cénat et al. 2018 | Canada | 34 Quebec high schools | 14-20 years | 1540 | 74.22% | \ | \ | - Cybervictimization   Psychological distressSubstance use |
| Dilmac et al. 2016 | Turkey | different secondary schools; a random cluster sampling method. | Secondary schools | 1743 | 55.00% | \ | \ | Peaceful, Honesty values  and Cyberbullying Sensibility |
| Festl, 2016 | Germany | a two-wave panel survey; seven schools in the southwest of Germany | Grades 7-10 | 1428 | 50.00% | \ | 25.00% Insulting:12.00%,  Visual violence:6.00%,  Used a fake identity: 6.00%,  Forwarded a private message:11.00% | - perpetrating cyberbullying   Perceived behavioral control  Previous perpetration of  Cyberbullying.  Direct peer influence for a person's close friends |
| Garaigordobil, 2015 | Spain | Basque Country; stratified, proportional, and randomized sampling | 12-18 years | 3026 | 51.50% | 30.20% Insulting messages:8.80%,  Insulting calls: 5.40%,  Anonymous frightening calls:9.80%,  Discredited:8.90%,  Password stolen 10.00%,  Identity theft:7.20% | 15.50% Insulting messages:5.20%, Insulting calls: 3.30%, Anonymous frightening calls:6.40%, Discredited:3.20%, Stealing a password:4.50%, Identity theft:2.50% | - Cybervictimization   Past experience of cyberaggression; cyberobservation; and aggressive-cybervictimization |
| Grinshteyn et al. 2017 | United States | the 2013 Youth Risk Behavior Survey (YRBS), a nationally representative survey of US high school students; A 3-stage cluster sample design; Weighting factors were applied to each record | Grades 9-12 | 13554 | \ | \ | \ | Absences |
| Ho et al. 2017 | Singapore | four primary schools and four secondary schools from four Singapore regions (North, South, East, and West)；Multi-stage cluster sampling | 9-17 years | 1424 | 47.60% | \ | \ | - Cyberbullying perpetration   Age,  Education level,  Attitudes,  Subjective norms,  Active mediation,  Restrictive mediation, |
| Hoareau et al. 2019 | France | 2 French public high schools | 11-15 years | 334 | 48.50% | \ | \ | Moral disengagement,  Psychopathy |
| Holfeld et al. 2017 | Canada | 27 predominantly rural schools across 3 provinces representing Eastern, Central, and Western Canada | Grades 5-6 | T1: n = 714；T2: n = 638 | 52.20% | \ | \ | - Cyber Victimization   School climate |
| Holt et al. 2016 | Singapore | a nationally representative sample of Singapore youth；8 secondary schools and 2 primary schools | Secondary school students and primary school students | 3226 | \ | \ | \ | - Cyberbullying Victimization   Home Internet use, OR=1.443*  Physical bullying, OR=1.269***  IM use, 1.235**  Bulletin board use, 1.200** |
| Hong et al. 2018 | South Korea | A nationally representative sample of South Korean adolescents；363 schools; systematic stratified  cluster sampling | Grades 4-12 | 10453 | 42.20% | \ | \ | - Direct cyberbullying victimization   Sex (male),  Neighborhood safety,  Family dysfunction,  Parental abuse,  Parental neglect,  Depression,  School victimization,  Poor peer relations   - Indirect cyberbullying victimization   Parental neglect,  Poor peer relations,  Teacher abuse,  School victimization,  Neighborhood safety, |
| Horzum et al. 2019 | Turkey | A city in northwestern Turkey; The stratified sampling method | 14-19 years | 1540 | 55.90% | \ | \ | Gender, t=4.901, p<0.001 Empathic tendency |
| Huang et al. 2019 | China | 24 primary, junior high and senior high schools from three cities in southern Taiwan; the method of convenient sampling | Elementary students, junior high school students and senior high school students | 1112 | 37.30% | 6%-31%  Insulting：5.30%, Mocking:5.10%, Being impersonated:1.10%, Revealing one's privacy without permission:1.20% | 6.00%-21.00% Insulting:3.80%, Disgracing:3.20%,  posting game account and password information without permission:1.30%, Impersonating to insult someone:2.80%, Impersonating for money:1.50% | - Victimization   Boys  Education level or age(high  school students)   - frequency and perceived   seriousness of bully/victim experiences  Education level or age(high  school students) |
| Iranzo et al. 2019 | Spain | in Valencia Region (Spain); stratified cluster sampling, with the sampling units being secondary schools. | 12-18 years | 1062 | 48.50% | \ | \ | - Cyberbullying Victimization   Perceived stress,  Loneliness,  Depressive symptomatology,  Psychological distress |
| Katz et al. 2019 | Israel | Regional middle school catering to mid to high SES families in the southern part of Israel. | Grades 7-8;  12-14.5 years | 180 | 49.44% | \ | \ | - Cyber bullying   Parents' general controlling style,  Cyber-specific inconsistent style, Cyber victimization  Parents' general controlling style,  Cyber-specific controlling style, Cyber-specific inconsistent style |
| Khurana et al. 2015 | United States | An online probability panel; probability-based (i.e., random-digit dialing and address-based) sampling | 12-17 years | 629 | 49.28% | \ | \ | - online harassment   Gender(female)Parental control, directly |
| Kim et al. 2018 | Canada | 248 schools; Stratification of the 180 communities by median family income (low, medium and high) resulted in over-sampling of schools from poorer and wealthier neighborhoods | Grades 6-12 | 31,148 | 52.20% | \ | \ | - Cyberbullying Victimization   For female, Emotional, For male, behavioral problems |
| Landoll et al. 2015 | United States | 2 high schools in a large metropolitan area in the Southeastern US | 14-18 years | 839 | 58.00% | \ | \ | - Cybervictimization   Depression, Social anxiety |
| Larranaga et al. 2016 | Spain | 4 public secondary schools in the Castilla-La Mancha region (Spain); A stratified sampling technique | Grades 7-10  12-18 years | 813 | 54.60% | \ | \ | - Cybervictimization   Loneliness  Offensive communication with mother |
| Lee et al. 2017a | South Korea | A national sample；24 middle and 24 high schools across South Korea; a multi-stage cluster sampling method; first, the country was stratified into 16 regions, including metropolitan areas such as Seoul and Pusan. Second, schools were selected in each region according to the proportion of the student population | Grades 7-12 | 4000 | 45.90% | 14.60%  be cyberbullied through a chat service:5.80%,  SNS service:3.40%,  Online game:10.20%,  Photograph/video:2.90%,  Text:2.80%,  Group violence:7.50%,  Disclosure of privacy:12.10%,  Be forced to run errands:1.20% | 6.30%  Cyberbullied someone through a chat service:7.00%,  SNS service:3.70%,  Online game:9.00%,  Photograph/video:1.00%,  Text:1.50%,  Group violence:10.10%,  Disclosure of privacy:1.70%,  Forced someone to run errands:0.90% | - cyberbullying perpetration   gender (male), OR=1.720***[95%CI: 1.353-2.186]  School (high school), OR=0.737** [95%CI: 0.607-0.894]  Cognitive empathy, OR=0.812 [95%CI: 0.705-0.936] |
| Lee, 2017b | United States | Northeastern and Midwestern United States | Grades 5-8 | 1096 | 49.00% | \ | \ | Weak commitment to school，  Deviant peers,  Past experience of cyberbullying victimization,  Differential association |
| Lin, 2016 | China | An elementary school in Taiwan, China | Grades 5-6;  11-13 years | 458 | 43.00% | 21.40% | 46.30% | Time spent online,  Parental supervision |
| Marco et al. 2018 | Spain | Several cities of Spain. | 12-19 years | 676 | 54.30% | 57.50% | \ | - Cyber victimization   Appearance evaluation,  Eating attitude test,  Overweight preoccupation, |
| Marret et al. 2017 | Malaysia | A national sample;  12 public secondary schools in the state of Negeri Sembilan; randomly selected | 15-16 years | 1487 | 53.90% | 52.20% Harassment:47.5%,  Sexual solicitation:19.10% | 32.00% Harassment:31.80%, Sexual solicitation：2.70% | Gender,  Environment |
| Martínez et al. 2019 | Spain | Public high school in middle class from neighborhoods in a Spanish city; A random selection of schools; a priori-determined sample size | 12-17 years | 1109 | 49.96% | \ | \ | - cyberbullying victimization   Authoritarian parenting style |
| Martinez-Ferrer et al. 2019 | Spain | 4 Compulsory Secondary Education (ESO) centres in the autonomous communities of Andalusia, Aragon and Valencia (Spain); random group sampling | 12-18 years | 1304 | 53.10% | \ | \ | - Direct Cyber-aggression   Parenting styles,  Gender |
| McQuillan, 2016 | United States | Public middle schools in a northeastern region of the United States; A convenience sampling method | Grades 6-8 | 1200 | 51.00% | \ | \ | - Cyberbullying   Age(high grade)   - Bully-Victim of Cyberbullying   Urban  Male  Age(high grade)  Physical or Psychiatric Condition  Parental Rejection   - Cyberbullying victim   Parental Rejection |
| Mesch, 2018 | United States | a nationally representative sample | 12-17 years | 800 | 56.40% | \ | \ | - Sharing password   Parental control, OR=0.84** |
| Moreno–Ruiz et al. 2019 | Spain | 19 public and private schools of Compulsory Secondary Education and High School of the provinces of Huelva, Seville, Cadiz, and Cordoba (western Andalusia, Spain); Stratified cluster sampling | 12-18 years | 2399 | 49.80% | \ | \ | Parenting styles |
| Morin et al. 2018 | United States | 58 Maryland high schools | Grades 9-12 | 28583 | \ | \ | \ | Grade(upperclassman), OR=1.188⁎⁎  Gender(male), OR=0.708⁎⁎  Race(Non-Caucasian), OR=0.955  Past experience of Cyber perpetrator, OR=5.242⁎⁎  Past experience of traditional victim, OR=3.346⁎⁎  Absence, OR=1.001 |
| Navarro et al. 2018 | Spain | Castilla-La Mancha region (Spain) | Grades 7-10 | 643 | 49.30% | \ | \ | Fatalism |
| Olenik-Shemesh et al. 2017 | Israel | 10 classes from 3 urban schools in the center of Israel | 14-16 years | 204 | 48.00% | 45.00% name calling ：20.00% insulting:14.00% threats:5.00% online harassment:18.00% Boycotted:5.00% Impersonating: 7.00% Sex harassment: 14.00% | \ | Gender,  Tradition bullying,  Self-esteem,  Social support, Self-efficacy |
| Olumide et al. 2016 | Nigeria | Schools in rural and urban LGA, Oyo state, Nigeria; A multi-stage technique | 12-16.5 years | 653 | 51.30% | 39.80% | 23.90% | - cyber-bullying   Online time(daily), OR=3.37 [95%CI:2.12–5.36]**  Past experience of cyberbullying victimization, OR=21.93 [95%CI: 13.02–36.93]**  Geographic location(urban), OR=1.48 [95%CI: 1.02–2.15]** |
| Pereira et al. 2016 | Portugal | 20 state schools and private schools in the Northern Region of Portugal and Azores; a stratified sampling | 12-16 years | 627 | 54.90% | Cyberstalking:62.00% | \ | Gender,  Social norms,  Past experience of bullying,  Romantic relationship |
| Pieschl et al. 2017 | Germany | 224 high school students most of whom attended grades 11 (n = 17), 12 (n = 123),  and 13 (n = 81) of grammar (n = 177), vocational  (n = 31), or comprehensive (n = 16) schools; 3  participants had already finished school | Grades 11-13 | 244 | 68.44% | 38.00% | 31.00% | Gender,  Past experience of cyberbullying,  Trust,  Parental control,  Parental-child relation,  Self-efficacy |
| Chen et al. 2018 | China | Using a two-staged  stratified sampling method, a total of 150 schools were first randomly sampled from Hong Kong and 5 cities in mainland China | Grades 9-12;  15-17 years | 18341 | 46.70% | \ | \ | - Internet Victimization   PTSD, OR=1.23***[95%CI: 1.091-1.394]  Depression, OR=1.05***[95%CI: 1.037-1.059]  Physical health OR=0.95*** [95%CI: 0.937-0.96)  Mental health, OR=0.99*[95%CI: 0.977-1]  Deliberate self-harm and suicide ideation OR=1.53*** [95%CI: 1.228- 1.914]  Father’s unemployment OR=2.07***[95%CI: 1.47, 2.908]  Gender(boy) OR=2.73*** [95%CI: 2.179, 3.411]  Family violence (lifetime) OR=2.79*** [95%CI: 2.19, 3.564] |
| Rao et al. 2019 | China | 6 different junior and senior high schools in Guangzhou, southern; using computer-generated random numbers | Grades 7-10 | 2590 | 40.30% | 44.50%， Flaming：32.00%， Denigration：17.10%， Harassment：20.20%， Exclusion：17.80%， Outing：11.30%，  Internet fraud：8.60%， cyberstalking：11.90% | 28.00%， Flaming22.10%， Harassment：6.90%， Denigration：5.80%， Exclusion：13.20%， Outing：5.00%， Internet fraud：3.90%， cyberstalking：5.00% | - Perpetrators   Online game addiction OR=4.478[95%CI: 1.567-12.800]**   - Victims   Parenting style of mother(No authoritative), OR=1.256 [95%CI: 1.011-1.560]**  Physical discipline by parents, OR=1.356[95%CI: 1.091-1.686)** |
| Razjouyan et al. 2018 | Iran | 10 junior high school students who were in the 11th grade in Tehran, Iran | 16-18 years | 505 | 42% | 34.20% | 27.30% | Gender |
| Reed et al. 2018 | United States | a self-report cross-sectional survey study of 9th to 12th grade students at a large Michigan suburban public high school campus with three high schools. | Grades 9-12 | 947 | \ | \ | \ | Gender |
| Rose et al. 2015 | United States | 12 midwestern schools | Grades 6-12 | 559 | 55.10% | \ | \ | Mental health |
| Sam et al. 2017 | United States | A nationally representative sample | 13-17 years | 215 | \ | \ | \ | The frequency of Internet use,  Social bonding,  Device use monitoring |
| Sarina et al. 2018 | Malaysia | 8 public schools around the Selangor state by using the  stratified random sampling technique | 9-16 years | 375 | 64.80% | \ | \ | Parental attachment,  Parent-child communication and trust,  Parental supervision |
| Sari, 2016a | Turkey | 3 different types of schools (science high school, Anatolian high school and public high school) | 15-17 years | 489 | 51.90% | \ | \ | Self-defeating humor |
| Sari et al. 2016b | Turkey | 3 different types of schools (science high school, Anatolian high school and public high school) | 15-17 years | 286 | 58,40% | \ | \ | Violence tendency |
| Sasson et al. 2017 | Israel | 13 different schools in a large city in Israel | Grades 6-11；  10-18 years | 495 | 46.00% | \ | \ | Gender,  Internet activity,  Peer influence |
| Simsek et al. 2019 | Turkey | All high schools in a center located in the Black Sea Region;  the stratified and simple random sampling methods | 15-17 years | 2422 | 48.50% | \ | \ | Internet use |
| Stockdale et al. 2015 | United States | A moderate-size community in the western US | 15-17 years | 106 | \ | \ | \ | Borderline personality disorder |
| Stoll et al. 2015 | United States | Bluffview High School | Grades 9-12 | 752 | 47.00% | \ | \ | Race |
| Tesler et al. 2019 | Israel | A nationally representative sample;  182 schools, 225 classrooms | Grades 6, 8, 10 | 7166 | 53.00% | 11.40% (secular students),  8.40% (religious  students) | \ | Age,  Gender,  Internet technology,  religion |
| Wang et al. 2016 | China | A middle school in Anhui, China | 12-14 years | 470 | 48.44% | \ | \ | Gender,  Moral disengagement |
| Wright, 2017 | United States | 10 middle schools were randomly selected a list of over 150 public middle schools located in the suburbs of a large Midwestern city | Grade 8 | 568 | \ | \ | \ | Parental mediation |
| Wright et al. 2015 | India | 6 private schools in the Karnataka state of India | 13-15 years | 480 | \ | \ | \ | Peer attachment |
| You et al. 2016 | South Korea | A nationally representative sample; a stratified multi-stage cluster sampling | 12-14 years | 3449 | 50.00% | \ | \ | - cyberbullying   perpetration  Bullying experience, OR=1.72⁎⁎⁎  Victim experience, 1.83⁎⁎  Computer use time on an average day, OR=1.12⁎⁎  lack of self-control, OR=1.37⁎⁎  Aggression, OR=1.31⁎⁎ |
| Yuan et al. 2019 | China | 6 high schools in east China | Grades 10-12 | 1274 | \ | \ | \ | Mindfulness,  Depression |
